# Serum creatinine trajectory after drainage of kidneys with bilateral malignant ureteral obstruction: a prospective non-randomized comparative study

**DOI:** 10.1186/s12894-023-01188-8

**Published:** 2023-02-22

**Authors:** Rabea Ahmed Gadelkareem, Ahmed Mahmoud Abdelraouf, Ahmed Mohammed El-Taher, Abdelfattah Ibrahim Ahmed, Mahmoud Mohamad Shalaby

**Affiliations:** Assiut Urology and Nephrology Hospital, Faculty of Medicine, Assiut University, Elgamaa Street, Assiut, 71515 Egypt

**Keywords:** Bilateral obstructed kidneys, Malignant ureteral obstruction, Percutaneous nephrostomy, Serum creatinine trajectory

## Abstract

**Background:**

Serum creatinine trajectory (SCr-Tr) is a neglected prognostic tool for chronic and acute kidney injury. We aimed to assess the predictors of SCr-Tr during the time-to-nadir and serum creatinine (SCr) normalization rate after drainage, using percutaneous nephrostomy in patients with bilateral malignant ureteral obstruction.

**Methods:**

A prospective non-randomized study was performed on SCr-Tr in patients with bilateral malignant ureteral obstruction from August 2019 to March 2022. The primary outcome was SCr-Tr during the time-to-nadir.

**Results:**

This study included 102 patients with a mean age ± SD of 59.6 ± 14.7 years. SCr-Tr was non-linear with a mean ± SD (range) of 0.5 ± 0.4 (0.03–2.3) mg/dl/day. Multivariate analyses revealed that female gender (*p* = 0.016), body mass index (BMI; *p* = 0.005), and SCr at presentation (*p* < 0.001) were predictors of rapid SCr-Tr during the time-to-nadir. However, age (*p* = 0.008) and low urine output at presentation (*p* = 0.015) were associated with a lower SCr-Tr. In contrast, laterality of drainage (*p* = 0.544) and mean parenchymal thickness (*p* = 0.066) were not associated with mean SCr-Tr. Also, only the mean parenchymal thickness (*p* = 0.002) was a predictor of rapid SCr-Tr at ≥ 0.5 mg/dl/day. However, low BMI (*p* = 0.023) was associated with a high SCr normalization rate, while unilateral drainage (*p* = 0.045) was associated with a lower rate.

**Conclusions:**

Female gender, low BMI, and SCr at presentation were predictors of rapid SCr-Tr during the time-to-nadir. Bilateral drainage was an independent predictor of SCr normalization rate, but not of rapid SCr-Tr. The mean parenchymal thickness was the only independent predictor for rapid SCr-Tr at ≥ 0.5 mg/dl/day.

## Background

Post-renal type of acute kidney injury (AKI) is an obstructive urinary tract pathology. The underlying causes of obstruction can be differentiated into benign and malignant ureteral obstruction (MUO) [1, 2]. The initial management of post-renal AKI is usually accomplished via a minimally invasive drainage of the bilateral obstructed kidneys. MUO has been reported as an independent predictor of the non-improvement of serum creatinine (SCr) after drainage [1, 3]. However, there is a lack of research in the literature on the serum creatinine trajectory (SCr-Tr) which is defined as the rate of changes in SCr concentrations over time [4]. The use of SCr-Tr may provide an attractive tool for monitoring of recovery of renal functions [5]. Because the baseline SCr of patients admitted to hospital with AKI is often lacking, management of those patients based on SCr-Tr rather than maximal SCr level may mitigate the need for the pre-admission baseline SCr [6]. In concordance, this issue generates a hypothesis that SCr-Tr can be associated with improvement in the management of post-renal AKI, regarding the pharmacotherapeutic adjustments or surgical interventions. Hence, we aimed to define the predictors of SCr-Tr as a sensitive tool for prognosis of management for normalization of SCr in patients with bilateral obstructed kidneys due to MUO.

## Methods

A prospective study was carried out at our hospital from August 2019 to March 2022. This study targeted patients with bilateral obstructed kidneys due to MUO. It was conducted according to the Transparent Reporting of Evaluations with Nonrandomized Designs (TREND) statement [7]. It included patients with age > 18 years, high SCr > 2 mg/dL, and bilateral MUO with grades 1–3 hydronephrosis according to the Onen grading system of hydronephrosis [8]. Exclusion criteria were bleeding tendency, severe comorbidity preventing intervention, non-simultaneous bilateral percutaneous nephrostomy (PCN), dialysis after drainage of obstruction, lost-to-follow-up patients, and refusal of participation in the study.

**Fig. 1:**
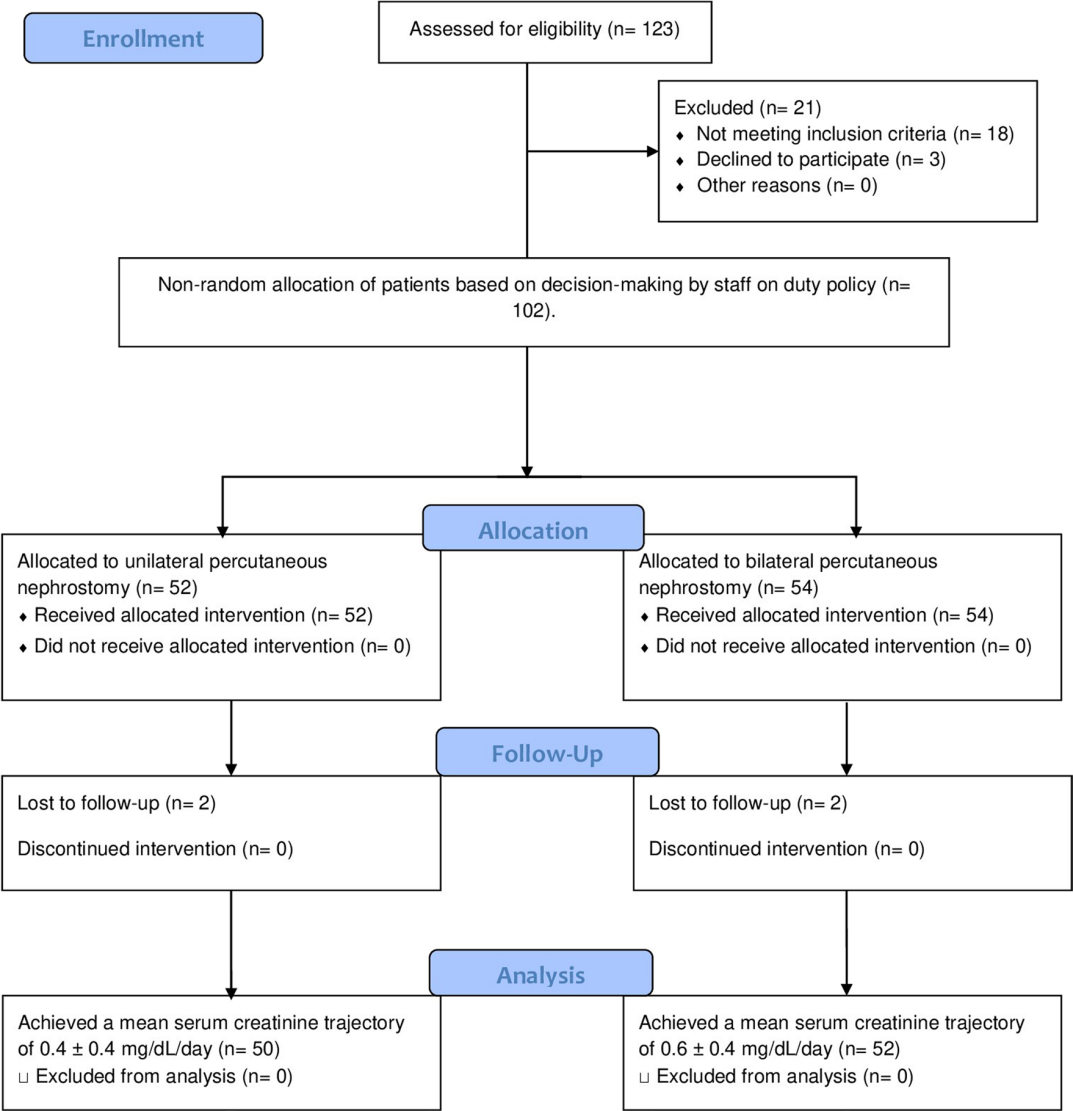
A flowchart of patients who underwent drainage of bilateral obstructed kidneys due to malignant ureteral obstruction showing the steps from assessment for eligibility, counseling, to non-random allocation to drainage intervention by unilateral or bilateral percutaneous nephrostomy, follow-up, and data analysis. Four patients were lost-to-follow-up. So, the actual number in both groups was 50 and 52 in unilateral and bilateral percutaneous nephrostomies groups, respectively, representing a total of 102 patients

The sample size was calculated using EasyMedStat version 3.17 (http://www.easymedstat.com), considering the power of the study 80%, a margin of error 10%, a confidence level of 95%, and a probability value of 0.5. A sample size of 96 patients was estimated. However, considering the percentage of patients with lost-to-follow-up, we enrolled 106 eligible patients. The number of patients who completed follow-up was 102 patients (Fig. 1). This study was conducted in accordance with the Declaration of Helsinki and its amendments. It was approved by the local ethical committee of the Faculty of Medicine, Assiut University and the institutional review board number is 17100860/2019, as a part of a study of bilaterally obstructed kidneys due to different etiologies. The latter was registered in ClinicalTrials: NCT04077008, 04/09/2019.

In all patients, a full history was taken, including a history of loin pain, uremic manifestations such as hiccough, vomiting, dyspepsia, anorexia, and urine output, comorbidity, and surgical interventions. Also, a systematic physical examination was performed, specifically for body temperature, body mass index (BMI), and loin tenderness. Laboratory workups included complete blood count, SCr, blood urea nitrogen, blood gases, and random blood sugar. In all cases, imaging studies included ultrasonography, kidney-ureter-bladder radiography, and computed tomography. Based on the inclusion criterion of grades of hydronephrosis, ultrasonographic measurement of the parenchymal thickness was estimated as the mean of the different thicknesses of the renal parenchyma at the different zones of the kidney.

Unilateral or bilateral drainage was conducted using PCN. Owing to the emergency, non-random allocation was due to the decision-making policy which was mostly dependent on the staff member on duty or the operator. In our center policy, the decision is usually made individually for unilateral and bilateral drainage. The emergency urologist decides the laterality of drainage, considering the general performance of the patient, anticipated effect on quality of life, and clinical and laboratory findings. In order, the priority of unilateral drainage is principally given for the infected, painful, or better parenchymal thickness kidney. However, bilateral drainage is warranted for expected bilateral infection or painful kidneys. Otherwise, the decision is individually tailored, based on the patient's conditions. Intraoperative and direct postoperative observations were performed for vital signs, consciousness level, and color and amount of urine. Finally, patients were discharged with instructions of healthcare within 1–3 days postoperatively.

The duration of follow-up of patients’ kidney function was planned over 3 weeks, on the first, second, third, fifth, tenth, fifteenth, and twenty-first days, postoperative. At each visit, patients were evaluated by physical examination, checking PCN patency, decompression of kidneys by ultrasonography, and SCr maximal level and SCr-Tr. The latter was defined as the rate of change of SCr level per day from its value at presentation of the patient to the lowest one within 21 days (time-to-nadir SCr).

The primary outcome of the study was the SCr-Tr during the time-to-nadir and it was defined at different values between 0.1 and 1 mg/dL/day during the time-to-nadir SCr after drainage of obstruction. Nadir SCr was defined as the decrease of SCr to a normal level or the lowest reading of SCr within 21 days. The secondary outcomes were the SCr normalization rate and time-to-nadir SCr. According to the mode of SCr-Tr at 0.3 and 0.5 mg/dL/day values, patients were further classified into those who had ≥ 0.3 or 0.5 mg/dL/day SCr-Tr (Rapid SCr-Tr group) and those who had < 0.3 or 0.5 mg/dL/day SCr-Tr (Slow SCr-Tr group). Due to the unknown value of the baseline SCr, it was considered the nadir SCr identified within 21 days of drainage. The normal SCr level was defined as 0.7–1.2 mg/dL and urine output was defined in different statuses as normal (> 400 ml/day) and low urine output; oliguria (100–400 ml/day) and anuria (< 100 ml/day). Complication grades were defined according to the modified Clavien-Dindo classification system [9].

Statistical analysis**:** It was performed using EasyMedStat (version 3.17; http://www.easymedstat.com). Numeric variables were expressed as mean ± standard deviation and categorical variables as counts and frequencies (%). We compared groups according to the laterality of drainage (Unilateral versus Bilateral PCN groups) and mode of SCr-Trs (Rapid versus Slow groups). Normality and heteroskedasticity of continuous data were assessed with Shapiro–Wilk and Levene’s tests, respectively. Continuous outcomes were compared with Mann–Whitney U, ANOVA, Welch ANOVA, or Kruskal–Wallis tests, according to data distribution. Categorical outcomes were compared with chi-squared test or Fisher’s exact test. Multivariate logistic or linear regression analyses were performed to assess the predictors of the mean SCr-Tr during time-to-nadir, modes of SCr-Tr, SCr level normalization rate, time-to-nadir, and their proposed explanatory variables. Data were checked for multicollinearity with the Belsley-Kuh-Welsch technique. A p-value of 0.05 or less was considered statistically significant.

## Results

After exclusion of 4 patients who were lost to follow-up, the current study included 102 patients who had a drainage for bilateral MUO, including 73 (71.6%) men and 29 (28.4%) women. Totally, the mean ± SD (range; interquartile range) of age {59.6 ± 14.7 (23–86; 53–70) year}, BMI {24.6 ± 4.8 (16.1–36.9; 20.6–27.6) kg/m^2^}, SCr level at presentation {6.3 ± 3.1 (2–17; 3.8–7.5) mg/dL}, SCr level at 3rd day {4.3 ± 2.6 (0.8–13; 2.2–5.5) mg/dL}, time-to-nadir SCr {12.6 ± 5.2 (3–21; 7–15) days}, and SCr-Trs during the first 3 days {0.7 ± 0.6 (0.2–0.9) mg/dL/day} and during the time-to-nadir {0.5 ± 0.4 (0.03–2.3; 0.2–0.6) mg/dL/day} were estimated.

In a comparison relative to the laterality of drainage by PCN, 50 patients received unilateral PCN and 52 patients received bilateral PCN. There were significant differences in age, urine output statuses at presentation, and SCr levels during the first to the third postoperative day. However, there were insignificant differences in all other variables, including SCr-Trs, SCr normalization rate, and time-to-nadir SCr (Table 1).

**Table 1:** A comparison between unilateral and bilateral PCN for bilateral obstructed kidneys with malignant ureteral obstruction

Variables	Unilateral PCN (n = 50)	Bilateral PCN (n = 52)	*p* value
	Mean ± SD (Range; IQR) at 95% CI or Number (Percentage)		
Age (year)	62.8 ± 13.7 (24–82; 58.9–66.7)	56.5 ± 15.3 (23–86; 52.3–60.8)	0.011
Gender			
Men	37 (74%)	36 (69.2%)	0.753
Women	13 (26%)	16 (30.8%)	
BMI (kg/m^2^)	24.4 ± 4.9 (16.1–36.9; 23–25.8)	24.7 ± 4.8 (17.3–35.9; 23.4–26.1)	0.618
Comorbidity			
Yes	17 (34%)	12 (23.1%)	0.588
None	33 (66%)	40 (76.9%)	
Smoking			
Yes	10 (20%)	13 (25%)	0.714
No	40 (80%)	39 (75%)	
Previous surgery			
Yes	5 (10%)	8 (15.4%)	0.654
None	45 (90%)	44 (84.6%)	
Pain			
Bilateral	17 (63%)	17 (60.7%)	0.789
Left	4 (14.8%)	6 (21.4%)	
Right	6 (22.2%)	5 (17.9%)	
Temperature	37.2 ± 0.5 (36.0–38.8; 37.0–37.3)	37.3 ± 0.4 (36.5–38.6; 37.1–37.4)	0.189
UOP status at presentation			
Normal	38 (76%)	27 (51.9%)	0.020
Low	12 (24%)	25 (48.1%)	
Tenderness			
Bilateral	4 (57.1%)	6 (60%)	> 0.999
Unilateral	3 (42.9%)	4 (40%)	
Pre dialysis sessions			
Once	7 (77.78%)	5 (41.67%)	0.184
More than one	2 (22.22%)	7 (58.33%)	
Uremic symptoms			
Present	7 (14%)	11 (21.2%)	0.711
Absent	43 (86%)	41 (78.8%)	
Mean parenchymal thickness (mm)	13 ± 2 (8.5–16; 12.4–13.6)	12.6 ± 2 (8–16;12–13.1)	0.249
Type of malignancy relative to urinary tract			
Urinary	34 (68%)	31 (59.6%)	0.500
Extraurinary	16 (32%)	21 (40.4%)	
Pathological type of malignancy			
Bladder cancer	27 (54%)	28 (53.8%)	0.663
Prostate cancer	7 (14%)	3 (5.8%)	
Colorectal cancer	9 (18%)	11 (21.2%)	
Cervical cancer	6 (12%)	8 (15.4%)	
Lymphoma	1 (2%)	2 (3.8%)	
Random blood sugar	124.8 ± 56.9 (45–330; 108.6–140.9)	126.9 ± 53 (72–315; 112.1–141.6)	0.529
Blood PH	7.37 ± 0.07 (7.18–7.47; 7.35–7.39)	7.36 ± 0.07 (7.15–7.47; 7.34–7.38)	0.448
PCO_2_ (mmHg)	25.1 ± 5.2 (15–36; 23.66–26.62)	24.1 ± 6.1 (14–36; 22.4–25.8)	0.249
Bicarbonate defect (mmol/L)	− 7.5 ± 5.4 {− 21 to 1; − 9.07–(− 5.99)}	− 9.13 ± 5.55 {− 21 to 1; − 10.67–(− 7.58)}	0.144
HCO_3_ (mmol/L)	15.8 ± 3.9 (9–23; 14.7–17)	15.7 ± 4.3 (6.7–22.5; 14.4–16.9)	0.825
SCr at presentation (mg/dL)	5.8 ± 3.2 (2–17; 4.9–6.7)	6.8 ± 3.1 (2.2–14; 5.9–7.6)	0.06
Postoperative SCr measures (mg/dL)			
SCr at 1st day	4.7 ± 2.8 (1.3–14.8; 3.9–5.5)	5.8 ± 2.7 (1.5–12.3; 5.1–6.5)	0.017
SCr at 3rd day	3.9 ± 2.7 (0.8–13; 3.1–4.6)	4.8 ± 2.5 (0.8–11; 4.1–5.5)	0.022
SCr at 5th day	3 ± 2 (0.6–8.2; 2.5–3.6)	3.7 ± 2.2 (0.7–9.1; 3.1–4.3)	0.119
SCr at 7th day	2.5 ± 1.6 (0.5–6.9; 2–3)	2.9 ± 2.1 (0.5–10.6; 2.3–3.5)	0.457
SCr at 10th day	2.1 ± 1.3 (0.3–5.2; 1.8–2.5)	2.2 ± 1.4 (0.3–4.4; 1.9–2.6)	0.683
SCr at 15th day	1.4 ± 0.7 (0.4–3.9; 1.2–1.6)	1.3 ± 0.7 (0.4–3.5; 1.1–1.5)	0.292
SCr at 21st day	1.4 ± 0.6 (0.4–2.5; 1.2–1.6)	1.2 ± 0.5 (0.4–2.5; 1.1–1.3)	0.101
Time-to-nadir SCr (days)	12.6 ± 5.2 (3–21; 11.1–14)	12.7 ± 5.4 (3–21; 11.2–14.2)	0.847
SCr normalization Rate			
Yes	24 (48%)	34 (65.4%)	0.116
No	26 (52%)	18 (34.6%)	
Pus cells in urine after drainage/HPF	27.8 ± 28.2 (5–100; 19.8–35.8)	30.8 ± 29.9 (3–100; 22.4–39.1)	0.432
SCr-Trs (mg/dL/day)			
SCr-Tr during 1st 3 days	0.7 ± 0.7 (− 1 to 3.2; 0.5–0.9)	0.7 ± 0.6 (− 0.3 to 3.3; 0.5–0.82)	0.656
Mode of SCr-Tr during 1st 3 days at 0.3 mg/dL/day value			
Rapid (≥ 0.3 mg/dL/day)	32 (64%)	37 (71.2%)	0.575
Slow(< 0.3 mg/dL/day)	18 (36%)	15 (28.8%)	
Mode of SCr-Tr during 1st 3 days at 0.5 mg/dL/day value			
Rapid (≥ 0.5 mg/dL/day)	24 (48.0%)	28 (53.85%)	0.695
Slow(< 0.5 mg/dL/day)	26 (52.0%)	24 (46.15%)	
Mode of SCr-Tr during 1st 3 days at 0.7 mg/dL/day value			
Rapid (≥ 0.7 mg/dL/day)	18 (36.0%)	19 (36.54%)	> 0.999
Slow(< 0.7 mg/dL/day)	32 (64.0%)	33 (63.46%)	
Mode of SCr-Tr during 1st 3 days at 1 mg/dL/day value			
Rapid (≥ 1 mg/dL/day)	11 (22%)	11 (21.2%)	> 0.999
Slow(< 1 mg/dL/day)	39 (78%)	41 (78.8%)	
SCr-Tr during 1st week	0.5 ± 0.4 (0.03–1.7; 0.4–0.6)	0.6 ± 0.4 (− 0.5 to 1.6; 0.4–0.7)	0.116
Mean SCr-Tr during time-to-nadir SCr (mg/dL/day)	0.4 ± 0.4 (0.03–2.1; 0.3–0.6)	0.6 ± 0.4 (0.1–2.3; 0.4–0.7)	0.076
Modes of SCr-Tr during time-to-nadir SCr at 0.1 mg/dL/day value			
Rapid (≥ 0.1 mg/dL/day)	47 (94%)	52 (100%)	0.114
Slow (< 0.1 mg/dL/day)	3 (6%)	0 (0%)	
Modes of SCr-Tr during time-to-nadir SCr at 0.3 mg/dL/day value			
Rapid (≥ 0.3 mg/dL/day)	30 (60%)	34 (65.4%)	0.721
Slow (< 0.3 mg/dL/day)	20 (40%)	18 (34.6%)	
Modes of SCr-Tr during time-to-nadir SCr at 0.5 mg/dL/day value			
Rapid (≥ 0.5 mg/dL/day)	15 (30%)	23 (44.2%)	0.2
Slow (< 0.5 mg/dL/day)	35 (70%)	29 (55.8%)	
SCr-Tr during 1st 3 days–SCr-Tr during 1st week (mg/dL/day)	0.2 ± 0.5 (− 1.3 to 1.7; 0.1–0.3)	0.1 ± 0.3 (− 0.4 to 1.7; 0.01–0.2)	0.284
SCr-Tr during 1st 3 days–SCr-Tr during time-to-nadir (mg/dL/day)	0.2 ± 0.5 (− 1.3 to 1.6; 0.1–0.4)	0.1 ± 0.3 (− 0.7 to 1.2; 0.0–0.2)	0.148

BMI: body mass index, SCr: serum creatinine, SCr-Tr: serum creatinine trajectory, UOP: urine output

On the other hand, comparing the patients according to their differentiation into rapid and slow SCr-Tr at different values revealed significantly different SCr levels and SCr-Trs during the first 3 days and within 21 days. At 0.3 mg/dL/day value, SCr levels were significantly different at the first postoperative 1–3 days and later after the tenth day, while they were insignificantly different during the third to tenth day. Also, SCr-Trs during the first 3 days and during the time-to-nadir SCr showed significant differences. Moreover, the SCr normalization rate was significantly higher in patients with rapid SCr-Tr during the time-to-nadir at a value ≥ 0.3 mg/dL/day (Table 2). At 0.5 mg/dl/day value, there were many differences from those at 0.3 mg/dL/day value in many variables, including urine output status at presentation, mean parenchymal thickness, types of underlying MUO, the timing of significant differences between the SCr levels and modes of SCr-Trs (Table 3).

**Table 2:** A comparison between Rapid (≥ 0.3 mg/dL/day) and Slow (< 0.3 mg/dL/day) groups of serum creatinine trajectory during the time-to-nadir

Variables	Rapid SCr trajectory (≥ 0.3 mg/dL/day) (n = 64)	Slow SCr trajectory (< 0.3 mg/dL/day) (n = 38)	*p* value
	Mean ± SD (Range; IQR) at 95% CI or Number (Percentage)		
Age (year)	58.3 ± 15.8 (24–86; 54.4–62.3)	61.7 ± 12.9 (23–82; 57.5–65.9)	0.544
Gender			
Men	41 (64.1%)	32 (84.2%)	0.051
Women	23 (35.9%)	6 (15.8%)	
BMI (kg/m^2^)	24.7 ± 4.7 (17.3–36.9; 23.5–25.9)	24.3 ± 5.1 (16.1–35.9; 22.6–26)	0.402
Smoking			
Yes	13 (20.3%)	10 (26.3%)	0.625
No	51 (79.7%)	28 (73.7%)	
Side of pain			
Bilateral	26 (68.4%)	8 (47.1%)	0.296
Right	6 (15.8%)	5 (29.4%)	
Left	6 (15.8%)	4 (23.5%)	
Temperature	37.3 ± 0.5 (36–38.8; 37.1–37.4)	37.1 ± 0.4 (36.5–38.6; 37–37.3)	0.083
UOP status at presentation			
Normal	37 (57.8%)	28 (73.7%)	0.162
Low	27 (42.2%)	10 (26.3%)	
Loin tenderness			
Unilateral	4 (30.8%)	3 (75%)	0.25
Bilateral	9 (69.2%)	1 (25%)	
Pre-drainage dialysis			
Once	9 (60%)	3 (50%)	> 0.999
More than once	6 (40%)	3 (50%)	
Mean parenchymal thickness (mm)	13 ± 1.9 (8–16; 12.5–13.5)	12.4 ± 2 (9.5–16; 11.7–13)	0.075
Pathological type of malignancy			
Bladder cancer	32 (50%)	23 (60.5%)	0.012
Prostate cancer	3 (4.7%)	7 (18.4%)	
Colorectal cancer	18 (28.1%)	2 (5.3%)	
Cervical cancer	9 (14.1%)	5 (13.2%)	
Lymphoma	2 (3.1%)	1 (2.6%)	
Type of malignancy relative to urinary tract			
Urinary	35 (54.7%)	30 (79%)	0.024
Extraurinary	29 (45.3%)	8 (21%)	
Random blood sugar	128.8 ± 54.7 (72–330; 115.1–142.4)	120.9 ± 54.9 (45–315; 102.8–138.9)	0.151
Blood PH	7.4 ± 0.1 (7.2–7.5; 7.3–7.4)	7.38 ± 0.05 (7.3–7.5; 7.4–7.4)	0.673
PCO_2_ (mmHg)	24.4 ± 5.4 (14–36; 23.1–25.8)	24.9 ± 6.1 (15–36; 22.9–26.9)	0.832
HCO_3_ (mmol/L)	15.2 ± 4.2 (6.7–23; 14.1–16.2)	16.7 ± 4 (11.2–23; 15.4–18)	0.128
Acid–base deficit (mmol/L)	− 9 ± 5.5 {− 21 to 1; − 10.34–(− 7.6)}	− 7.3 ± 5.5 (− 15 to 1; − 9.1 to 5.5)	0.316
Laterality of drainage			
Unilateral	30 (46.9%)	20 (52.6%)	0.721
Bilateral	34 (53.1%)	18 (47.4%)	
SCr at presentation (mg/dl)	7.5 ± 3.4 (2–17; 6.6–8.3)	4.3 ± 1 (2.2–6.4; 4–4.7)	< 0.001
Post-drainage SCr measures (mg/dl)			
SCr at 1st day	6.1 ± 3.1 (1.3–14.8; 5.3–6.8)	3.9 ± 1.1 (1.9–6.2; 3.6–4.3)	< 0.001
SCr at 3rd day	4.8 ± 3.2 (0.8–13; 4–5.6)	3.5 ± 1.1 (1.4–5.7; 3.2–3.9)	0.157
SCr at 5th day	3.5 ± 2.6 (0.6–9.1; 2.8–4.1)	3.2 ± 1 (1.5–5.3; 2.8–3.5)	0.685
SCr at 7th day	2.7 ± 2.3 (0.5–10.6; 2.1–3.2)	2.7 ± 1 (1–5.6; 2.4–3.1)	0.28
SCr at 10th day	2.1 ± 1.5 (0.3–5.2; 1.7–2.4)	2.4 ± 0.8 (1.2–3.9; 2.1–2.6)	0.122
SCr at 15th day	1.3 ± 0.75 (0.4–3.5; 1.1–1.5)	1.6 ± 0.55 (0.8–3.9; 1.4–1.7)	0.010
SCr at 21st day	1.2 ± 0.62 (0.4–2.5; 1–1.3)	1.5 ± 0.43 (0.8–2.5; 1.4–1.6)	0.001
Time-to-nadir SCr (days)	11 ± 5.8 (3–21; 9.5–12.4)	15.4 ± 2.3 (7–21; 14.7–16.2)	< 0.001
SCr normalization rate during 21 days			
Normal SCr	42 (65.6%)	16 (42.1%)	0.035
High SCr	22 (34.4%)	22 (57.9%)	
Pyuria at 1 week after drainage	28.5 ± 28.6 (5–100; 21.4–35.7)	30.61 ± 29.9 (3–100; 20.8–40.5)	0.934
Pus cells in urine after drainage /HPF	28.5 ± 28.6 (5–100; 21.4–35.7)	30.6 ± 29.9 (3–100; 20.8–40.5)	0.934
SCr-Trs (mg/dL/day)			
SCr-Tr during 1st 3 days	0.9 ± 0.7 (− 1–3.3; 0.7–1.1)	0.3 ± 0.2 (0–0.7; 0.2–0.3)	< 0.001
Mode of SCr-Tr during 1st 3 days at 0.3 mg/dL/day value			
Rapid SCr-Tr (≥ 0.3 mg/dL/day)	54 (84.4%)	15 (39.5%)	< 0.001
Slow SCr-Tr (< 0.3 mg/dL/day)	10 (15.6%)	23 (60.5%)	
Mode of SCr-Tr during 1st 3 days at 0.5 mg/dL/day value			
Rapid SCr-Tr (≥ 0.5 mg/dL/day)	48 (75%)	4 (10.5%)	< 0.001
Slow SCr-Tr (< 0.5 mg/dL/day)	16 (25%)	34 (89.5%)	
Mode of SCr-Tr during 1st 3 days at 0.7 mg/dL/day value			
Rapid SCr-Tr (≥ 0.7 mg/dL/day)	37 (57.81%)	0 (0.0%)	< 0.001
Slow SCr-Tr (< 0.7 mg/dL/day)	27 (42.2%)	38 (100%)	
Mode of SCr-Tr during 1st 3 days at 1 mg/dL/day			
Rapid SCr-Tr (≥ 1 mg/dL/day)	22 (34.4%)	0 (0%)	< 0.001
Slow SCr-Tr (< 1 mg/dL/day)	42 (65.6%)	38 (100%)	
SCr-Tr during 1st week	0.68 ± 0.39 (− 0.53 to 1.7; 0.58–0.78)	0.23 ± 0.15 (− 0.19 to 0.57; 0.18–0.28)	< 0.001
Mean SCr-Tr during time-to-nadir SCr	0.68 ± 0.42 (0.3–2.34; 0.57–0.78)	0.18 ± 0.067 (0.03–0.29; 0.16–0.21)	< 0.001
Mode of SCr-Tr during time-to-nadir SCr at 0.1 mg/dL/day value			
Rapid SCr-Tr (≥ 0.1 mg/dL/day)	64 (100%)	35 (92.1%)	0.049
Slow SCr-Tr (< 0.1 mg/dL/day)	0 (0%)	3 (7.9%)	
Mode of SCr-Tr during time-to-nadir at 0.5 mg/dL/day value			
Rapid SCr-Tr (≥ 0.5 mg/dL/day)	38 (59.4%)	0 (0%)	< 0.001
Slow SCr-Tr (< 0.5 mg/dL/day)	26 (40.6%)	38 (100%)	
SCr-Tr during 1st 3 days-SCr-Tr during 1st week (mg/dL/day)	0.21 ± 0.5 (− 1.33 to 1.7; 0.09–0.33)	0.05 ± 0.13 (− 0.2 to 0.36; 0.004–0.09)	0.079
SCr-Tr during 1st 3 days–SCr-Tr during time-to-nadir SCr (mg/dL/day)	0.2 ± 0.5 (− 1.28 to 1.56; [0.09–0.34)	0.09 ± 0.15 (− 0.17 to 0.51; 0.04–0.14)	0.478

BMI: body mass index, SCr: serum creatinine, SCr-Tr: serum creatinine trajectory, UOP: urine output

**Table 3:** A comparison between Rapid (≥ 0.5 mg/dL/day) and Low (< 0.5 mg/dL/day) groups of serum creatinine trajectory during the time-to-nadir

Variables	Rapid SCr trajectory (≥ 0.5 mg/dL/day) (n = 52)	Slow SCr trajectory (< 0.5 mg/dL/day) (n = 50)	*p* value
Age (year)	57.4 ± 15.1 (24–77; 53.2–61.6)	61.9 ± 14.2 (23–86; 57.9–65.9)	0.253
Gender			
Men	37 (71.2%)	36 (72%)	> 0.999
Women	15 (28.8%)	14 (28%)	
BMI (kg/m^2^)	24.6 ± 4.8 (17.3–36.9; 23.2–25.9)	24.6 ± 4.9 (16.1–35.9; 23.2–26)	0.820
Comorbidity			
Yes	33 (63.5%)	31 (62%)	0.898
No	19 (36.5%)	19 (38%)	
Smoking			
Yes	13 (25%)	10 (20%)	0.714
No	39 (75%)	40 (80%)	
Pain			
None	23 (44.2%)	24 (48%)	0.181
Right	5 (9.6%)	6 (12%)	
Left	3 (5.8%)	7 (14%)	
Bilateral	21 (40.4%)	13 (26%)	
Temperature	37.3 ± 0.5 (36.5–38.8; 37.1–37.4)	37.2 ± 0.5 (36–38.6; 37–37.3)	0.213
UOP status at presentation			
Normal	22 (57.89%)	43 (67.19%)	0.465
Low	16 (42.11%)	21 (32.81%)	
Tenderness			
Unilateral	4 (36.4%)	3 (50%)	0.644
Bilateral	7 (63.6%)	3 (50%)	
Pre-drainage dialysis			
Once	8 (50%)	1 (20%)	0.338
More than once	8 (50%)	4 (80%)	
Uremic symptoms			
Yes	16 (30.8%)	13 (26%)	0.263
No	36 (69.2%)	37 (74%)	
Mean parenchymal thickness (mm)	13.6 ± 1.6 (9–16; 13.1–14.1)	12.3 ± 2 (8–16; 11.8–12.8)	< 0.001
Pathological type of malignancy			
Bladder cancer	26 (50%)	29 (58%)	0.119
Prostate cancer	3 (5.8%)	7 (14%)	
Colorectal cancer	15 (28.9%)	5 (10%)	
Uterine cervical cancer	6 (11.5%)	8 (16%)	
Lymphoma	2 (3.8%)	1 (2%)	
Type of malignancy relative to urinary tract			
Urinary	19 (50%)	46 (71.9%)	0.045
Extraurinary	19 (50%)	18 (28.1%)	
Random blood sugar	134.1 ± 62.3 (75–330; 116.8–151.4)	117.3 ± 44.4 (45–280; 104.6–130)	0.18
Blood PH	7.36 ± 0.09 (7.15–7.47; 7.34–7.39)	7.37 ± 0.05 (7.23–7.47; 7.367.39)	0.96
PCO_2_ (mmHg)	24.9 ± 5 (14–36; 23.5–26.3)	24.3 ± 6.3 (15–36; 22.5–26.1)	0.411
Acid–base deficit (mmol/L)	− 8.5 ± 5.8 {(− 21)− 1; (− 10.1)–(− 6.9)}	− 8.2 ± 5.3 {(− 20)–1; (− 9.7)–6.7}	0.771
HCO_3_ (mmol/L)	15.5 ± 4.3 (9–23; 14.3–16.7)	16 ± 4 (6.7–23; 14.8–17.1)	0.475
Laterality of drainage			
Unilateral	24 (46.2%)	26 (52%)	0.695
Bilateral	28 (53.8%)	24 (48%)	
SCr at presentation (mg/dL)	7.8 ± 3.4 (2.6–17; 6.9–8.8)	4.7 ± 1.8 (2–9.2; 4.2–5.2)	< 0.001
Post-drainage SCr measures (mg/dL)			
SCr at 1st day	6.1 ± 3.2 (1.3–14.8; 5.2–7)	4.4 ± 1.8 (1.7–9; 3.9–4.9)	0.013
SCr at 3rd day	4.6 ± 3.2 (0.8–13; 3.7–5.5)	4.1 ± 1.9 (1.2–9.8; 3.5–4.6)	0.997
SCr at 5th day	3.2 ± 2.4 (0.6–8.2; 2.5–3.8)	3.5 ± 1.8 (0.9–9.1; 3–4)	0.112
SCr at 7th day	2.4 ± 1.9 (0.5–6.5; 1.9–2.9)	3 ± 1.8 (0.5–10.6; 2.5–3.5)	0.044
SCr at 10th day	1.9 ± 1.4 (0.3–5; 1.5–2.3)	2.5 ± 1.2 (0.5–5.2; 2.1–2.8)	0.021
SCr at 15th day	1.2 ± 0.7 (0.4–2.9; 1–1.4)	1.6 ± 0.68 (0.5–3.9; 1.4–1.7)	0.007
SCr at 21st day	1.1 ± 0.6 (0.4–2.5; 1–1.3)	1.5 ± 0.5 (0.5–2.5; 1.3–1.6)	< 0.001
Time-to-nadir SCr (days)	11.3 ± 6 (3–21; 9.7–13)	14 ± 4 (5–21; 12.9–15.1)	0.014
SCr normalization rate during 21 days			
Normal SCr	37 (71.2%)	21 (42%)	0.006
High SCr	15 (28.8%)	29 (58%)	
Pus cells in urine after drainage/ HPF	24.9 ± 26.3 (6–100; 17.6–32.2)	33.9 ± 31.1 (3–100; 25–42.7)	0.169
SCr-Trs (mg/dL/day)			
Mean SCr-Tr during 1st 3 days	1.1 ± 0.6 (0.5–3.3; 0.9–1.3)	0.2 ± 0.2 {(− 1)–0.5; 0.2–0.3)}	< 0.001
Mode of SCr-Tr during 1st 3 days at 0.3 mg/dL/day value			
Rapid (≥ 0.3 mg/dL/day)	52 (100%)	17 (34%)	< 0.001
Slow (< 0.3 mg/dL/day)	0 (0%)	33 (66%)	
Mode of SCr-Tr during 1st 3 days at 0.7 mg/dL/day value			
Rapid (≥ 0.7 mg/dL/day)	37 (71.2%)	0 (0%)	< 0.001
Slow (< 0.7 mg/dL/day)	15 (28.8%)	50 (100%)	
Mode of SCr-Tr during 1st 3 days at 1 mg/dL/day value			
Rapid (≥ 1 mg/dL/day)	22 (42.3%)	0 (0%)	< 0.001
Slow (< 1 mg/dL/day)	30 (57.7%)	50 (100%)	
SCr-Tr during 1st week	0.8 ± 0.4 (0.2–1.7; 0.7–0.9)	0.2 ± 0.2 (− 0.5 to 0.8; 0.2–0.3)	< 0.001
Mean SCr-Tr during time-to-nadir SCr	0.7 ± 0.4 (0.2–2.3; 0.6–0.9)	0.2 ± 0.1 (0–0.8; 0.2–0.3)	< 0.001
Mode of SCr-Tr during time-to-nadir SCr at 0.1 mg/dL/day value			
Rapid (≥ 0.1 mg/dL/day)	52 (100%)	47 (94%)	0.114
Slow (< 0.1 mg/dL/day)	0 (0%)	3 (6%)	
Mode of SCr-Tr during time-to-nadir at 0.3 mg/dl/day value			
Rapid (≥ 0.3 mg/dL/day)	48 (92.31%)	16 (32.0%)	< 0.001
Slow (< 0.3 mg/dL/day)	4 (7.69%)	34 (68.0%)	
Mode of SCr-Tr during time-to-nadir SCr at 0.5 mg/dl/day value			
Rapid (≥ 0.5 mg/dL/day)	37 (71.15%)	1 (2.0%)	< 0.001
Slow (< 0.5 mg/dL/day)	15 (28.85%)	49 (98.0%)	
SCr-Tr during 1st 3 days-SCr-Tr during 1st week (mg/dL/day)	0.3 ± 0.5 (− 0.37 to 1.7; 0.2–0.5)	− 0.03 ± 0.2 (− 1.3 to 0.2; − 0.1 to 0.04)	< 0.001
SCr-Tr during 1st 3 days–SCr-Tr during time-to-nadir (mg/dL/day)	0.4 ± 0.5 (− 0.3 to 1.6; 0.2–0.5)	− 0.04 ± 0.3 (− 1.3 to 0.3; − 0.1 to 0.04)	< 0.001

BMI: body mass index, SCr: serum creatinine, SCr-Tr: serum creatinine trajectory, UOP: urine output

In multivariate linear regression analysis, female gender (*p* = 0.016), low BMI (*p* = 0.005), and SCr level at presentation (*p* < 0.001) were associated with higher mean SCr-Tr during the time-to-nadir. However, age (*p* = 0.008) and low urine output at presentation (*p* = 0.015) were associated with lower mean SCr-Tr during the time-to-nadir. On the other hand, laterality of drainage (*p* = 0.544) and mean parenchymal thickness (*p* = 0.066) were not associated with the mean SCr-Tr during time-to-nadir (Table 4).

**Table 4:** A multivariate linear regression analysis of the predictors of the serum creatinine trajectory during the time-to-nadir

Variables	Odds ratio	*p* value
Age (year)	− 0.01 [− 0.01; − 0.002]	0.008
Gender	0.26 [0.05; 0.46]	0.016
BMI (kg/m^2^)	0.02 [0.01; 0.04]	0.005
Urine output status at presentation	− 0.17 [− 0.30; − 0.03]	0.015
Mean parenchymal thickness (mm)	0.03 [− 0.002; 0.06]	0.066
Type of malignancy relative to urinary tract	− 0.17 [− 0.39; 0.051]	0.130
Laterality of drainage	− 0.04 [− 0.16; 0.08]	0.544
SCr level at presentation (mg/dL)	0.08 [0.06; 0.11]	< 0.001

BMI: body mass index, SCr: serum creatinine

Multivariate logistic regression analyses of the predictors of the modes of SCr-Tr at 0.5 mg/dL/day during the time-to-nadir revealed that only the mean parenchymal thickness (*p* = 0.002) was associated with rapid SCr-Tr at 0.5 mg/dL/day (Table 5).

**Table 5:** Multivariate logistic regression analysis of the predictors of the mode of serum creatinine trajectory during the time-to-nadir defined at 0.5 mg/dL/day rate

Variables	Odds ratio	*p* value
Age (year)	0.98 [0.95; 1.01]	0.149
Urine output	1.24 [0.49; 3.14]	0.647
Mean parenchymal thickness (mm)	1.51 [1.17; 1.94]	0.002
Laterality of drainage	0.50 [0.19; 1.29]	0.152

In multivariate linear regression analysis, age (*p* = 0.031) and low urine output at presentation (*p* = 0.040) were associated with longer time-to-nadir. However, the female gender (*p* = 0.012) was associated with shorter time-to-nadir. In contrast, the mean parenchymal thickness (*p* = 0.257) and laterality of drainage (*p* = 0.912) were not associated with the time-to-nadir (Table 6).

**Table 6:** A multivariate linear regression analysis of the predictors of the time-to-nadir duration

Variables	Coefficients	*p* value
Age (year)	0.11 [0.01; 0.21]	0.031
Gender	− 3.72 [− 6.61; − 0.83]	0.012
Body mass index (Kg/m^2^)	− 0.21 [− 0.44; 0.02]	0.067
Urine output status at presentation	2.09 [0.09; 4.09]	0.040
Mean parenchymal thickness (mm)	− 0.29 [− 0.78; 0.21]	0.257
Type of malignancy relative to urinary tract	1.82 [− 1.58; 5.22]	0.292
Acid–base deficit (mmol/L)	− 0.12 [− 0.3; 0.06]	0.193
Laterality of drainage	− 0.12 [− 2.03; 1.81]	0.912
Pyuria after drainage (Pus cells/HPF)	− 0.015 [− 0.05; 0.02]	0.359

In multivariate logistic regression analysis, low BMI (*p* = 0.023) was associated with higher rates of SCr normalization. However, unilateral drainage (*p* = 0.045) was associated with lower rates (Table 7).

**Table 7:** A multivariate logistic regression analysis of the predictors of the serum creatinine normalization rate

Variables	Odds Ratio	*p* value
Gender	0.91 [0.31; 2.69]	0.864
BMI (kg/m^2^)	1.14 [1.02; 1.27]	0.023
Urine output status at presentation	0.74 [0.27; 1.99]	0.546
Mean parenchymal thickness (mm)	1.14 [0.90; 1.43]	0.283
Laterality of drainage	0.40 [0.16; 0.98]	0.045
SCr level at presentation (mg/dL)	0.97 [0.84; 1.13]	0.713

BMI: body mass index, SCr: serum creatinine

Regarding the complications, there was postoperative hematuria in 4 patients in each group. They were treated conservatively without blood transfusion (Grade 2). PCN slippage occurred only in 2 patients and were repositioned within 3–5 h (Grade 3).

## Discussion

In bilateral obstructed kidneys due to MUO, drainage is mostly achieved by placement of PCN until stabilization of the renal functions that is usually monitored by tracing the decrease of the maximal level of SCr. Many factors may predict the recoverability of renal function, including the laterality of drainage. These predictors have variably been studied without reaching a consensus on the optimal strategy and its prognostic values for drainage of bilateral obstructed kidneys [3, 10].

On the other hand, the classification of AKI based on SCr-Tr considers the patient’s response to early therapeutic interventions using the information provided by serial measures of SCr. Thus, the identification of AKI responses by SCr-Tr might improve the precision of risk stratification and provide more homogeneous groups of AKI cases [6]. Our study targeted SCr-Tr in patients with post-renal AKI after initial drainage by PCN. We believed that this intervention should be justified on sound prognostic bases to differentiate which case will get benefited. We adjusted the primary outcome as the mean SCr-Tr with specific values ≥ 0.3 mg/dL/day because the latter has been used to classify the stages of AKI [3, 6].

SCr-Tr during the first 3 days after drainage showed rapid patterns of reduction. They were significantly different between the groups of SCr-Trs at 0.3 and 0.5 mg/dL/day values, when the latter were differentiated into rapid and slow modes. Also, there were significantly different SCr-Trs during the time-to-nadir. However, there were insignificant differences between the values of the magnitude of the change between the first 3 days and time-to-nadir SCr-Trs. This refers to similar non-linear declines in both modes of SCr-Tr at two values on which the mode of SCr-Tr was classified. The non-linear declines could be explained by the effects of the significantly different SCr levels at the presentation on the SCr-Tr in the early days. This early measurement of SCr-Tr could be used as an indicator of the mode of recovery of AKI [4]. Also, it might be due to the effect of the underlying malignancy before SCr normalization or nadir. However, there was no significant effect for the laterality of drainage on SCr-Tr at 0.3 mg/dL/day value. On the other hand, the SCr normalization rate was significantly different between the modes of SCr-Tr at these values.

The differences between SCr-Trs during the first 3 days after the drainage and those during the time-to-nadir were insignificant, referring to parallel patterns of SCr-Trs in both groups. This effect happened, despite the insignificantly different effect of laterality of drainage on SCr normalization rates and SCr-Trs during the time-to-nadir. The latter, however, was significantly longer in patients with slow SCr-Tr than that in patients with rapid SCr-Tr. Also, it remained longer than the known values in previous studies of patients with benign ureteral obstructions [2]. Moreover, SCr normalization rate was significantly higher with rapid than with slow SCr-Tr.

In parallel, the current results revealed that SCr levels in groups of SCr-Tr were significantly different only on the first postoperative day and later, after the tenth day. However, they were insignificantly different during the days from the third to tenth day. This non-linear pattern reflects the same effect of high SCr at presentation on the mean values of SCr-Trs. However, it may also reflect the superiority of measuring the SCr-Tr on the maximal SCr levels in the evaluation of the cases of post-renal AKI [4, 6].

The SCr at presentation had no statistically significant effect on the SCr normalization rate, which was similar to many previous studies [11–13]. However, it was different from the previous results found that low SCr at presentation was a significant predictor for the recoverability of renal functions in patients with post-renal AKI [14–16]. Again, the significant effect of SCr at presentation was obvious on SCr-Trs referring to the superiority of the latter to maximal SCr levels.

According to the current study, bilateral drainage of MUO had a statistically significant effect on the SCr normalization rate. Although previous studies have reported a significant effect of bilateral drainage on the recovery of renal functions [1, 15], our results showed limited effects of laterality of drainage on the values of SCr levels represented only on the first postoperative day.

There are no guidelines addressing the recommendation for the preferences of laterality of drainage [1, 3, 17]. Some studies found no significant effect of drainage laterality to reach a nadir SCr reporting a time-to-nadir SCr of 7.7–10 days [11, 17]. In the current study, the time-to nadir was planned to be best measured within 21 days. The reasoning for using this cut-off time duration was based on multiple principles. Firstly, similar values have frequently been reported for the time-to-nadir in the literature with a range of 1–3 weeks [1, 11, 17, 18]. Secondly, we aimed to avoid the effects of other potential confounding factors of prolonged indwelling urinary catheters such as infections, slippage and obstruction which become inevitable and influence the effectiveness of drainage and SCr stabilization [19, 20]. Thirdly, the obstructions in the current study were mostly acute and included only patients with mild-to moderate degrees of hydronephrosis. In addition, and as we defined the improved SCr, the proposed outcome was to reach the lowest SCr level rather than the normal levels. This concept represents a common attitude in the recently reviewed literature. The importance and main aim of reduction of SCr should be avoidance of the inconveniences of dialysis and the balance between this benefit and the disturbance of quality of life of those patients [1, 20].

Bladder cancer has been identified as a cardinal etiology of post-renal AKI due to MUO, where genitourinary malignancies represent variable proportions up to 25% [1, 15, 21, 22]. Similarly, our results showed that the main underlying causes were urologic tumors. Moreover, other studies reported that improved patients with MUO would need a longer time to improve to normalize up to > 15 days [1, 18], which was comparable to our results that showed a mean time-to-nadir of 12.6 ± 5.2 days.

To our knowledge, previous studies of bilateral MUO have not considered studying SCr-Trs or their therapeutic and prognostic values. Hence, the current study may represent a motivator for further investigation of this subject.

The limitations of the current study included the non-randomized allocation of patients in receiving unilateral versus bilateral drainage. Also, studying patients’ survival and quality of life were not within the scope of this study.

## Conclusions

Female gender, low BMI, and SCr level at presentation were predictors of higher mean SCr-Tr during the time-to-nadir, but age and low urine output at presentation were associated with lower values. Bilateral drainage was an independent predictor for the SCr normalization rate, but not for rapid SCr-Tr. The mean parenchymal thickness was the only independent factor of rapid SCr-Tr at 0.5 mg/dL/day. Age and low urine output at presentation were associated with longer time-to-nadir. However, the female gender was associated with shorter time-to-nadir. In contrast, the mean parenchymal thickness and laterality of drainage were not associated with the value of the time-to-nadir. With further studying and more strong evidence, we may suggest utilization of SCr-Tr for stratification and differentiation of those patients, especially those cases with an expected favorable prognosis of the underlying malignancies.

## Data Availability

The data used and analyzed during the current study are available from the corresponding author on reasonable request.

## Abbreviations

AKI: Acute kidney injury
BMI: Body mass index
MUO: Malignant ureteral obstruction
PCN: Percutaneous nephrostomy
SCr: Serum creatinine
SCr-Tr: Serum creatinine trajectory
